# Deciphering the impact of exogenous fatty acids on *Listeria monocytogenes* at low temperature by transcriptome analysis

**DOI:** 10.3389/fmicb.2024.1441784

**Published:** 2024-09-04

**Authors:** Aurore Quilleré, Maud Darsonval, Angelos Papadochristopoulos, Alban Amoros, Pierre Nicolas, Florence Dubois-Brissonnet

**Affiliations:** ^1^Université Paris-Saclay, INRAE, AgroParisTech, MICALIS Institute, Jouy-en-Josas, France; ^2^Université Paris-Saclay, INRAE, MaIAGE, Jouy-en-Josas, France

**Keywords:** foodborne pathogen, refrigeration, exogenous fatty acids, fatty acid membrane composition, transcriptomics, RT-qPCR, flagella, *cheY* gene

## Abstract

*Listeria monocytogenes* is a ubiquitous and psychrotrophic foodborne pathogen commonly found in raw materials, ready-to-eat products, and food environments. We previously demonstrated that *L. monocytogenes* can grow faster at low temperature when unsaturated fatty acids (UFA) are present in its environment. This could question the maintenance of food safety for refrigerated foods, especially those reformulated with a higher ratio of UFA versus saturated fatty acids (SFA) to fit with nutritional recommendations. In this study, we used transcriptomics to understand the impact of UFA on the behavior of *L. monocytogenes* at low temperature. We first demonstrated that *fabK*, a key gene in SFA synthesis, is up-regulated in the presence of UFA but not SFA at low temperature. *L. monocytogenes* can thus regulate the synthesis of SFA in its membrane according to the type of FA available in its environment. Interestingly, we also observed up-regulation of genes involved in chemotaxis and flagellar assembly (especially *cheY* and *flaA*) in the presence of UFA but not SFA at low temperature. TEM observations confirmed that *L. monocytogenes* acquired a remarkable phenotype with numerous and long-looped flagella only in the presence of UFA at 5°C but not at 37°C. As flagella are well known to be involved in biofilm formation, this new finding raises questions about the structure and persistence of biofilms settled in refrigerated environments using unsaturated lipid-rich products.

## 1 Introduction

Listeriosis, caused by the foodborne pathogen *Listeria monocytogenes*, is the fifth most reported zoonosis and has a high fatality rate (13.7% in Europe, in 2021) (European Food Safety Authority and European Centre for Disease Prevention and Control, 2022). *L. monocytogenes* is ubiquitous and can be found in a wide variety of foodstuffs, widely present in food-associated environments (raw materials, food chain, and retail environments) leading to a high prevalence of this bacterium in foodstuffs. Ready-to-eat (RTE) products are consumed without cooking or other processing that could remove or reduce pathogens to acceptable levels. For this food category, which can support the growth of *L. monocytogenes*, EC Regulation No. 2073/2005 established that no more than 100 CFU/g should be found in 5 samples at the end of shelf-life. During shelf-life, *L. monocytogenes* growth is mainly controlled by refrigeration temperature.

The cold adaptation response is a common mechanism, well-described in psychrotrophic bacteria such as *Bacillus cereus* and *L. monocytogenes* (Alvarez-Ordóñez et al., 2015). Low temperature directly impacts cell integrity and basal cell functions including membrane fluidity, nutrient uptakes, protein folding and assembly of macromolecules (Zhu et al., 2005; Tasara and Stephan, 2006). The cold-related loss of membrane fluidity is due to a membrane phase transition from liquid-crystalline to gel state (Denich et al., 2003). The first transient adaptive response to low temperature is the regulation of the fatty-acid synthesis type II (FASII) for increasing the proportion of FA with lower melting points into the phospholipids and restoring optimal membrane fluidity (Annous et al., 1997).

The FA composition of *L. monocytogenes* mainly consists of *iso*- or *anteiso*-branched fatty acids (*i-*BFA and *a-*BFA) (84% of the membrane FA at 37°C in TSB) (Touche et al., 2023). BFA are produced from valine (*i*-C14, *i*-C15), leucine (*i*-C15, *i*-C17), and isoleucine (*a*-C15, *a*-C17) by transamination and decarboxylation to form acyl-CoA derivatives that enter then in the elongation module (Zheng et al., 2005). The FA profile of *L. monocytogenes* also contains saturated (SFA) and unsaturated (UFA) fatty acids at smaller proportions (13 and 3%, respectively at 37°C in TSB) (Touche et al., 2023). SFA are produced from acetate through the initiation and elongation modules. Although the presence of UFA in its membrane was previously described, as was the increase of their proportion when temperature decreases, the pathway of UFA synthesis has not been yet identified (Hingston et al., 2017; Touche et al., 2023). Two pathways for UFA synthesis are described in other bacterial species: (1) *de novo* synthesis through the FabM or FabN pathway; or (2) desaturation of an acyl chain through desaturase activity. As far as we know, none of them have been described in *L. monocytogenes*. The FabM or FabN pathway, described respectively in *Streptococcus* spp. and *Enterococcus faecalis*, converts *trans*-2-enoyl-ACP into acyl-ACP with a double bond leading to UFA synthesis (Fujita et al., 2007; Dong and Cronan, 2022). This FabM/N pathway enters in competition with the FabK pathway leading to SFA synthesis. The regulation between these two pathways modulates the SFA/UFA ratio and thus membrane fluidity. Therefore, if this pathway exists in *L. monocytogenes*, the bacteria could sustain optimum membrane fluidity by modulating the ratio UFA/SFA, besides the classical modulations of the membrane fluidity in Gram-positive bacteria (ratios of BFA/SFA, *a*-BFA/*i*-BFA, length of the FA acyl chain (Annous et al., 1997; Tasara and Stephan, 2006)).

Moreover, *L. monocytogenes* was recently shown to incorporate exogenous FA (#FA) into its membrane (Flegler et al., 2022; Touche et al., 2023). Direct incorporation of #FA could save the energy cost of *de novo* FA synthesis. Touche et al. (2023) showed that the incorporation of #FA into the membrane of *L. monocytogenes* is temperature-dependent but non-selective. All #FA are highly incorporated into the membrane at low temperature, but not at 37°C, and the effect on bacterial growth depends on the type of FA: #UFA promote and #SFA inhibit bacterial growth. This phenomenon is of major importance in terms of food safety. Indeed, today’s nutritional and health challenges are to reduce the ratio of SFA/UFA in the consumers’ diet. Consequently, food operators tend to follow these recommendations and reformulate their products in this way in the last 20 years. However, the increase of UFA in refrigerated foods can favor the growth of *L. monocytogenes* and can compromise food safety before the end of shelf-life (Touche et al., 2023).

The mechanisms of FA incorporation and its effects on general metabolism are still unknown. Gram-positive bacteria, such as *Staphylococcus aureus* and *Streptococcus* spp., can incorporate #FA through a FakAB system working as a two-component system (TCS). FakA, a FA kinase, phosphorylates FakB, the FA-binding protein, which in turn transfers the phosphate to the FA. The resulting acyl-phosphate is then transferred to acyl-carrier protein (ACP) by PlsX, the acyltransferase (Parsons et al., 2014). Different FakB chains with differences in selectivity have been described: FakB1 exclusively binds SFA, FakB2 binds SFA and mono-UFA, and FakB3 binds poly-UFA (Gullett et al., 2019). As far as we know, this mechanism is still not described in *L. monocytogenes*.

In this study, we first analyzed the global transcriptome of *L. monocytogenes* at 5°C and 37°C with or without oleic acid to identify the genes associated with the observed impact of UFA on *L. monocytogenes* grown at low temperature (RNA-seq). Targeted transcriptomic analysis (RT-qPCR) and phenotypic characterizations (FA membrane profiles and TEM observations) were then conducted with different types of #FA and at different growth phase points to further decipher the impact of #FA on the metabolism and behavior of *L. monocytogenes* at low temperature.

## 2 Materials and methods

### 2.1 Bacterial strain and culture conditions

*Listeria monocytogenes* CNL895805, shortly named Lm208, used in this study was isolated from sheep brain in France (serotype 1/2a, CC7, BioSample SAMN39851069) (Tabouret et al., 1992; Van Langendonck et al., 1998; Touche et al., 2023). This strain was chosen among nine *L. monocytogenes* strains of different origins because of its highest overgrowth capabilities in the presence of #UFA at low temperature (Touche et al., 2023). It was stored in Tryptone Soy Broth (TSB, bioMérieux, Marcy l’Etoile, France) supplemented with 20% (v/v) glycerol at −80°C (Invitrogen^™^, Thermo Fisher Scientific, Waltham, United States). The strain was inoculated at 1% v/v in TSB (≈10^6^ CFU/mL) with a standardized inoculum obtained after two successive subcultures at 30°C without shaking. When indicated, the medium was supplemented with different #FA, namely myristic acid (#C14), oleic acid (#C18:1), and linoleic acid (#C18:2) (Larodan Fine Chemicals, Malmö, Sweden). These #FA solutions were prepared as previously described (Touche et al., 2023) and the final FA concentration in the culture medium was 0.045 mM FA in 0.05% bovine serum albumin (w/v) (BSA, Sigma-Aldrich, Merck KGaA, Darmstadt, Germany). For RNA sequencing, *L. monocytogenes* was grown with #C18:1 or without and incubated at 5°C or 37°C. For RT-qPCR, membrane FA analysis, and TEM observations, *L. monocytogenes* was grown with #C14, #C18:1, #C18:2 or without at 5°C.

### 2.2 Cell harvest at different time points

Bacterial growth was followed by measuring optical density (OD) at 600 nm (Genesis 30 spectrophotometer, Thermo Fisher Scientific, Villebon-sur-Yvette, France) and cells were harvested at different time points: the mid-exponential phase at OD_600_ = 0.1 ± 0.05 (T1), the end of the exponential phase at OD_600_ = 0.4 ± 0.05 (T2) and the beginning of the stationary phase at OD_600_ = 0.7 ± 0.05 (T3). Cells were harvested at T1 for RNA sequencing and at T1, T2, and T3 for RT-qPCR and TEM observations. The harvested volume was adjusted to collect 10^9^ cells (100 mL for T1, 10 mL for T2, and 5 mL for T3). For RNA extraction, cells were harvested by centrifugation at 10,000 rpm for 2 min at the temperature of the culture when the target OD_600_ was reached. The pellet was then immersed in liquid nitrogen and stored at −80°C before extraction. For TEM observations, cells were harvested by centrifugation at 3,000 rpm for 10 min to preserve cell integrity and fixed immediately.

### 2.3 RNA extraction

Total RNA was extracted by the Trizol^™^ reagent method (Invitrogen^™^, Thermo Fisher Scientific) with some adaptations (Toledo-Arana et al., 2009). Pellets were resuspended in 1 mL of Trizol^™^ and transferred into a screw-cap tube containing 0–50 μm glass beads for cell disruption by bead beating for 40 s at 6 m/s (FastPrep-24 instrument, MP Biomedicals, Illkirch, France). RNA contained in the upper aqueous phase was washed with chloroform, precipitated with cold isopropanol, and finally washed twice with 75% ethanol (Sigma Aldrich). The RNA pellet was dried for 30 s at 63°C and resuspended in 20 μL of nuclease-free water (Invitrogen^™^, Thermo Fisher Scientific). Total RNA was treated by DNaseI and purified using the Monarch^®^ RNA Cleanup kit (New England BioLabs^®^ Inc., Evry-Courcouronnes, France). Before RNA sequencing and RT-qPCR, Nanodrop (2000 NanoDrop^™^ spectrophotometer, Thermo Fisher Scientific) and Qubit^™^ (Qubit^™^ 3 Fluorometer with the Qubit^™^ RNA HS assay kit, Invitrogen^™^, Thermo Fisher Scientific), respectively, were used to quantify RNA. Samples with a concentration above 10 ng/μL were used. RNA quality was assessed with a BioAnalyzer (Agilent 2100 Bioanalyzer System with RNA 6000 Nano kit, Agilent, Santa Clara, United States) and Nanodrop (2000 NanoDrop^™^ spectrophotometer, Thermo Fisher Scientific). Samples with an RNA Integrity Number (RIN) above 7.5 were used for RNA sequencing. Samples with A_260/280_ and A_260/230_ ratios above 2.0 and 1.8, respectively, were used for the RT-qPCR. Three to six biological replicates were performed for each of the conditions.

### 2.4 RNA sequencing and data analysis

RNA sequencing was performed by the I2BC platform (Paris-Saclay University, Gif-sur-Yvette, France, https://www.i2bc.paris-saclay.fr/) on an Illumina NextSeq sequencer NS500446. Primary data processing was performed by the I2BC platform with the following data analysis pipeline: demultiplexing (with bcl2fastq2 v.2.18.12), adapter trimming (with Cutadapt v.1.15), quality control (with FastQC v.0.11.5) resulting in 20 M to 34 M reads per sample, post QC and trimming. Reads were mapped to the Lm208 genome (BioProject PRJNA1074122, SRR29855365) using the BWA 0.6.2-r126. This generated between 8 M and 13 M uniquely mapped reads per sample. Mapping efficiency ranged from 77.86 to 84.34% for individual reads. FeatureCounts (v.1.5.2) was used to assign sequence reads to genomic features. The downstream data was analyzed using the R programming language with the Rstudio interface (v.4.1.2) and R package DESeq2 (v.1.30.1) (Love et al., 2014) based on the raw counts provided by the I2BC platform. First, 83 genes with fewer than 10 counts in the three biological replicates were excluded. Then, raw counts were normalized according to gene length and the total number of mapped reads by calculating FPKM (Fragments per Kilobase per Million Mapped reads) using the library-size estimation method implemented in R package DESeq2 (v.1.30.1) (Love et al., 2014). These FPKM values were used as expression level values for each gene in each condition. To assess the overall reproducibility, normalized counts of samples (log2(fpkm+5)) were compared by computing pairwise Pearson coefficients (*r*) and distances (1 − *r*). The distances were computed by average-linkage clustering using the “hclust” function (v.3.6.2) and summarized by an ascending hierarchical classification.

### 2.5 Clustering of gene expression profile

To detect differentially expressed genes (DEG) between pairs of conditions, log2 fold-change (log2FC) and *p*-values (*p*) were calculated using DESeq2. Four pairwise comparisons were considered: “FA5.vs.C5”; “FA37.vs.C37”; “C5.vs.C37”; “FA5.vs.FA37” where “FA” and “C” refer to the conditions with #C18:1 or without (control), respectively, and “5” and “37” refer to the culture temperature in °C. To control the false discovery rate of each pairwise comparison, the R package “fdrtool” (v.1.2.17) (Strimmer, 2008) with the vector of *p*-values was used to estimate *q*-values (*q*). The DEG reported for the four pairwise comparisons were based on a *q*-value ≤0.05 and |log2FC| ≥1. The expression profiles of the DEG across all samples were compared by computing pairwise Pearson distances (on log2(fpkm+5)) and subjected to average-link hierarchical classification with “hclust” function (v.3.6.2).

Clusters of DE genes, called DE No. (1 to 35), were defined by cutting the hierarchical clustering tree at a Pearson distance of 0.3 (average Pearson correlation above 0.7). Clusters were labeled according to their size, DE1 being the largest cluster. The resulting dendrogram and heatmap were produced with the “heatmap.2” function included in the R package “gplots” (v.3.1.1), using a specific color palette with red for up-regulated genes (*q* ≤ 0.05 and log2FC ≥1) and blue for down-regulated genes (*q* ≤ 0.05 and log2FC ≤−1). The whole transcriptomic dataset has been deposited in GEO (accession number GSE268246).

### 2.6 Functional categorization of differentially expressed genes

Functional categories were assigned to the genes of DE clusters using the Listeriomics database (Bécavin et al., 2017) and the SmartTables tool based on the BioCyc database^1^ (Caspi et al., 2016) with *Listeria monocytogenes* EGD-e genome as a reference. For each DE cluster, the percentage of functional categories was calculated and is summarized in Supplementary Table S1.

### 2.7 RT-qPCR and data analysis

Primers were designed using Primer-Blast^2^ (Table 1) with the following settings: a PCR amplicon size between 100 and 120 pb in the first third of the coding sequence, a melting temperature between 57.0 and 63.0°C with an optimum of 60°C and a difference of 3°C. Primer pair efficiency was assessed by performing the qPCR reaction on serial dilutions of gDNA extracted from Lm208 (64, 32; 16; 8 and 1.6 ng/μL). The efficiency was assessed twice with independent gDNA dilutions and calculated as described by Pfaffl (2001). Primer pairs with an efficiency above 1.8 were selected for RT-qPCR. The reference gene was chosen among six genes selected for their stable expression in all conditions of the transcriptomic analysis and tested for stability in RT-qPCR. Stability was assessed twice with or without different #FA (#C14, #C18:1, or #C18:2) at 5°C by calculating a stability *M* value using the geNorm module integrated in the qbase+ software (v.3.4). The smaller the *M* value (below 0.5) the greater the gene stability: *lmo208__02104* (*lmo2262* in *L. monocytogenes* EGD-e, hypothetical protein), which had the smallest *M* value (*M* = 0.120 and 0.157 for the two replicates), was selected as reference gene (Vandesompele et al., 2002; Hellemans et al., 2007). For each sample, 1 μg of total RNA was reverse transcribed using the QuantiTect Reverse Transcription Kit (Qiagen, Hilden, Germany) to obtain cDNA. cDNA was diluted at 1:10 and qPCR was conducted in a StepOne^™^ Real-Time PCR System (Thermo Fisher Scientific) using SsoAdvanced^™^ Universal SYBR Green^®^ Supermix (BioRad, Hercules, United States) with 250 nM for each primer. The transcript levels of genes of interest were normalized to the mean of the transcript levels of *lmo208__02104* (reference gene) in all conditions. The relative expression was calculated using the comparative 2^−ΔΔCT^ method by calculating ∆_CT_ and ∆∆_CT_ according to the two formulas below (Livak and Schmittgen, 2001):


ΔCT=CTCgene of interest−CTCreference geneΔΔCT=ΔCTC#FA−ΔCTCcontrol

The relative expression levels of each gene in the different conditions with #C14, #C18:1, or #C18:2 were compared to the control without #FA at each time point. The control is fixed to a relative expression level of 1.

**Table 1:** Primers used for quantitative PCR.

Lm208 locus tag	Gene name	Product	Primers 5′ → 3′		Amplicon size (bp)	Efficiency
*lmo208__02104*	NA^*^	Hypothetical protein	F	TGGCTATTTACTTGCGCAACG	100	2.064
			R	TGTAGCCTGGTCTTCCGGAT		
*lmo208__00517*	*cheY*	Chemotaxis response regulator	F	GCGGAAAATGGACTGGAAGC	105	1.978
			R	CGCAAGTGCTTCTAAGCCATC		
*lmo208__00516*	*flaA*	Flagellin	F	TGATGACGCTGCTGGTCTTG	119	1.989
			R	GAGCTGAATCCGCTGTTTGT		
*lmo208__02013*	*fabK2*	Enoyl-ACP-reductase	F	TACAAGGGGCAATGGCACAA	102	2.002
			R	TGTCTGCTGACATTCCACCA		
*lmo208__02015*	*pct*	Propionate CoA transferase	F	GGGCTCCGCGTAATCTTACA	115	1.898
			R	GCAATTCCACCAATCCAGCG		
*lmo208__02018*	*fabG*	3-ketoacyl-ACP-reductase	F	GGCGCAAAAGTAGTAGTGGC	105	1.918
			R	TACATTAGCTGCGACGGCAA		

^*^NA, not applicable.

### 2.8 Membrane fatty acid analysis

Bacterial pellets were washed twice with 0.1% Triton X-100 to remove unincorporated #FA. FA extraction and methylation were performed as previously described (Dubois-Brissonnet et al., 2016; Touche et al., 2023). Briefly, FA were saponified and methylated with methanolic NaOH and methanolic HCl solutions (1st step: 1 mL 3.75 M NaOH in 50% v/v methanol solution for 30 min at 100°C; 2nd step: addition of 2 mL 3.25 M HCl in 45% v/v methanol solution for 10 min at 80°C). FA methyl esters (FAME) were extracted with a diethyl ether/cyclohexane solution (1:1 v/v), and the organic phase was finally washed with a dilute NaOH solution (0.3 M NaOH). Reagents were purchased from Sigma Aldrich, Merck KGaA. Analytical gas chromatography of FAME was carried out on a GC-MS Trace 1300/ISQ 7000 (Thermo Fisher Scientific) equipped with a BPX70 capillary column (25 m, 0.22 mm id) (SGE^™^, Victoria, Australia). The column temperature was set at 100°C for 1 min and then increased to 170°C at the rate of 2°C/min. FAME were expressed as a percentage of the total area and grouped into classes: saturated fatty acids (SFA), unsaturated fatty acids (UFA), *iso-and anteiso*-branched-chain fatty acids (*i*-BFA and *a*-BFA).

### 2.9 Transmission electron microscopy

Bacterial pellets were fixed for 1 h at room temperature with 2% glutaraldehyde (Delta Microscopies, Mauressac, France) and centrifuged at 3,000 rpm for 10 min at 5°C. Cells were resuspended in 0.1 M cacodylate buffer (Delta Microscopies) and stored at 4°C (maximum 3 weeks). Cells were loaded onto a carbon film membrane on a 300-mesh copper grid which was rinsed twice with 0.1 M cacodylate buffer and stained with 1% uranyl acetate (Delta Microscopies). Finally, the grid was observed with a Hitachi HT7700 80 kV transmission electron microscope (TEM) at the MIMA2 platform (Paris-Saclay University, INRAE, AgroParisTech, GABI, 78350 Jouy-en-Josas, France, https://www6.jouy.inrae.fr/mima2/). Images were acquired with a charge-coupled device (CDD) camera (AMT Imaging, Woburn, United States). Cell and flagella lengths were measured on all the original micrographs using NIH ImageJ software (V1.54d). In each image, the total flagellar length was measured using the free-hand line tool and reported to the number of bacteria in the image to obtain an average flagellar length per bacterium.

### 2.10 Statistics

Statistical analysis was performed using GraphPad Software (v.10.1.2, Prism, United States). All experiments were performed with independent subcultures and the number of replicates is specified in the legend of each figure. Two-ways ANOVA with Tukey’s multiple comparison test (95% confidence interval) was performed on the data of RT-qPCR and fatty acid composition. One-way ANOVA with Tukey’s multiple comparison test (95% confidence interval) was performed on the data of bacterial cell and flagella lengths. According to the *p*-value, results were reported as significantly different *if *p* < 0.0332; ** if *p* < 0.0021; *** if *p* < 0.0002; **** if *p* < 0.0001.

## 3 Results

### 3.1 Global assessment of the *Listeria monocytogenes* transcriptome

**Figure 1:**
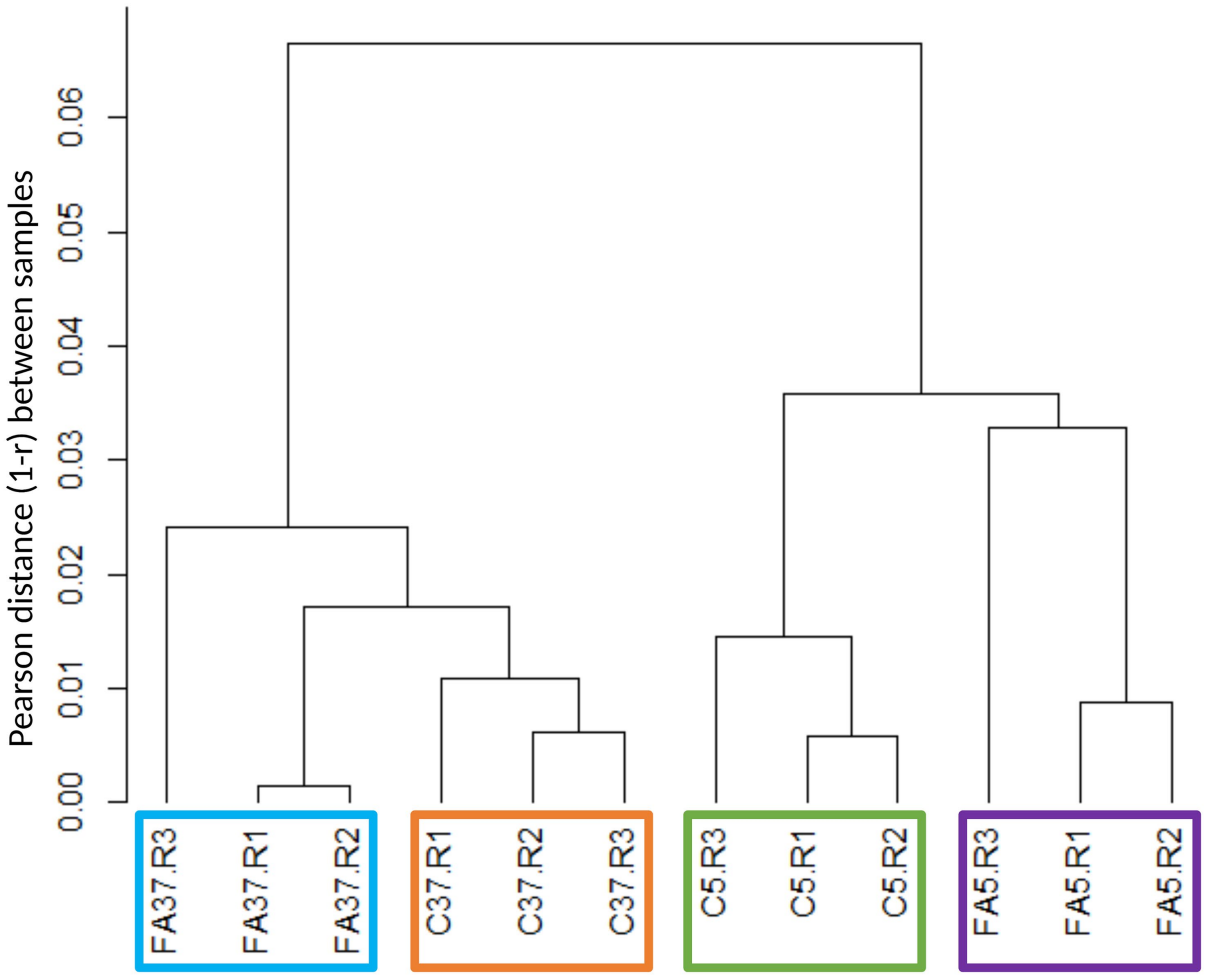
Hierarchical clustering tree of RNA sequencing biological samples obtained by calculating the Pearson coefficients per pair (*r*) and represented through the Pearson (1 − *r*). C indicates control cultures, FA indicates cultures grown with #C18:1, 5 and 37 refer to the culture temperature in °C. R1, R2 and R3 are the three replicates of each condition.

20 M to 34 M of high quality paired reads were generated and assigned to the genome of Lm208. Of 2,879 genes, 97.1% were expressed (83 genes with fewer than 10 counts in the three biological replicates in all conditions were excluded). Ascending hierarchical clustering of the samples was performed to assess the quality and reproducibility of the RNA-seq data (Figure 1; Supplementary Figure S1). The resulting dendrogram reveals that samples are first divided by the impact of temperature, and then by the impact of the presence of #C18:1. According to Pearson distance, replicates with #C18:1 were more heterogenous than control samples at each temperature. The presence of #C18:1 had a greater effect at 5°C than at 37°C.

**Figure 2:**
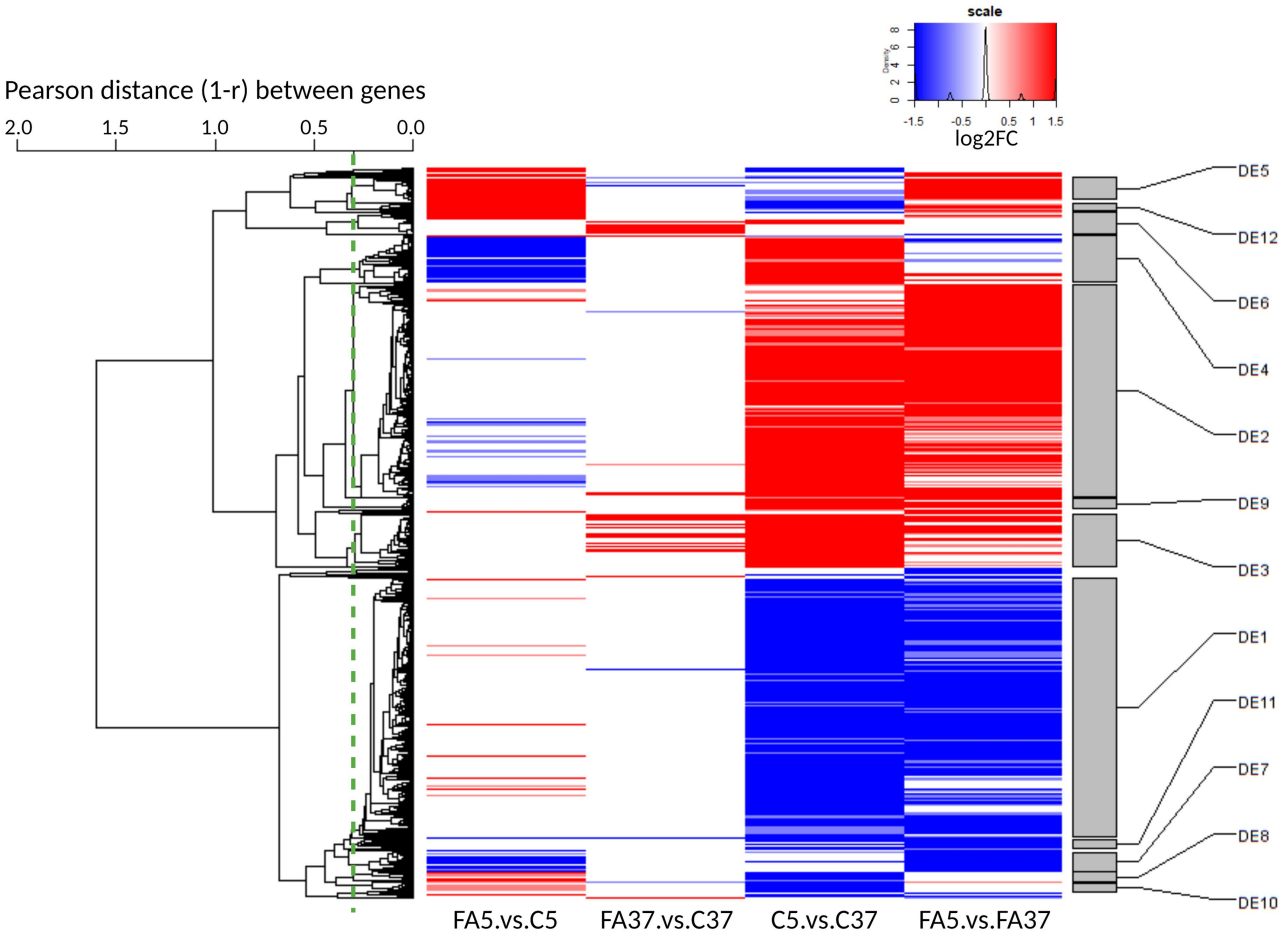
Global heatmap representation of the relative variations of expression level across 4 pairwise comparisons (C and FA refer to control cultures and cultures grown with #C18:1, 5 and 37 refer to the culture temperature) for differentially expressed genes. The dendrogram on the left side of the heatmap represents the hierarchical clustering of the 1,068 gene expression profiles across all samples. The dendrogram was cut at an average Pearson correlation of 0.7 (dashed green line) to define the differential expression (DE) clusters on the right side of the heatmap. Clusters were named from DE1 to DE35 by decreasing size. Only clusters with more than 10 genes are represented. Up-regulated genes are reported in red (*q* ≤ 0.05 and log2FC ≥1) and down-regulated genes in blue (*q* ≤ 0.05 and log2FC ≤ −1).

Of all genes, 1,068 were differentially expressed in at least one of the four pairwise comparisons considering a *q*-value ≤0.05 and |log2FC| ≥1 (Figure 2). To explore the impact of oleic acid at low temperature, we mainly focused on the pairwise comparison FA5.vs.C5: 94 and 88 genes were up-and down-regulated, respectively. Moreover, the impact of the temperature without #FA was investigated with the pairwise comparison C5.vs.C37: 408 and 414 genes were up-and down-regulated, respectively. The comparison FA37.vs.C37 allowed us to point out the impact of #FA at 37°C: 62 and 6 genes were up-and down-regulated, respectively. Finally, the comparison FA5.vs.FA37 compared the temperature effect in the presence of #C18:1: 337 and 341 genes were up-and down-regulated, respectively.

The DEG were grouped according to the expression profiles of the four pairwise comparisons into 35 DE clusters. In the 12 DE containing more than 10 genes (Figure 2), the three largest clusters, DE1, DE2, and DE3, presented more than half of the 1,068 DEG, with 377, 312, and 77 genes, respectively. These genes were mainly involved in general bacterial metabolism. In contrast, in the other DE, the genes were mainly involved in more specific pathways with one or two major functional categories (Supplementary Table S1). The genes in DE1 were down-regulated at low temperature. They include a cold shock protein (*cspB*), a propionate-CoA-transferase (*lmo2172* called here *pct*) involved in lipid transport and metabolism, and genes involved in virulence (*prfA*, *plcA*, *hly*, *actA*, and *plcB*). In the presence of #C18:1 at low temperature, *cspB* and *lmo2172* were up-regulated, with a log2FC = 1.43 and 2.26, respectively, but genes involved in virulence, as most of the genes from DE1, showed no significant difference in their expression profile. The genes clustered in DE2 and DE3 were up-regulated at low temperature. DE2 included *cspA* and genes encoding for transporters involved in cryoprotection (*opuCABCD*, and *gbuABC* genes). DE3 included genes involved in general function and carbohydrate metabolism (*glpK* and *glpD*). With #C18:1 at low temperature, most of the genes from these three DE showed no significant difference in their expression profiles.

30.4% of DE4 genes are involved in inorganic ion transport and metabolism, such as the genes *tatAC*, *hbp1* and *hbp2*, *isdGEF*, *efeOBU*, and *fhuCBG*. The genes in DE4 were up-regulated at low temperature, mostly with a log2FC >2, but only in the absence of #C18:1 (C5.vs.C37). In the presence of #C18:1 at low temperature (FA5.vs.C5), these genes were also down-regulated, mostly with a log2FC < −2.

**Figure 3:**
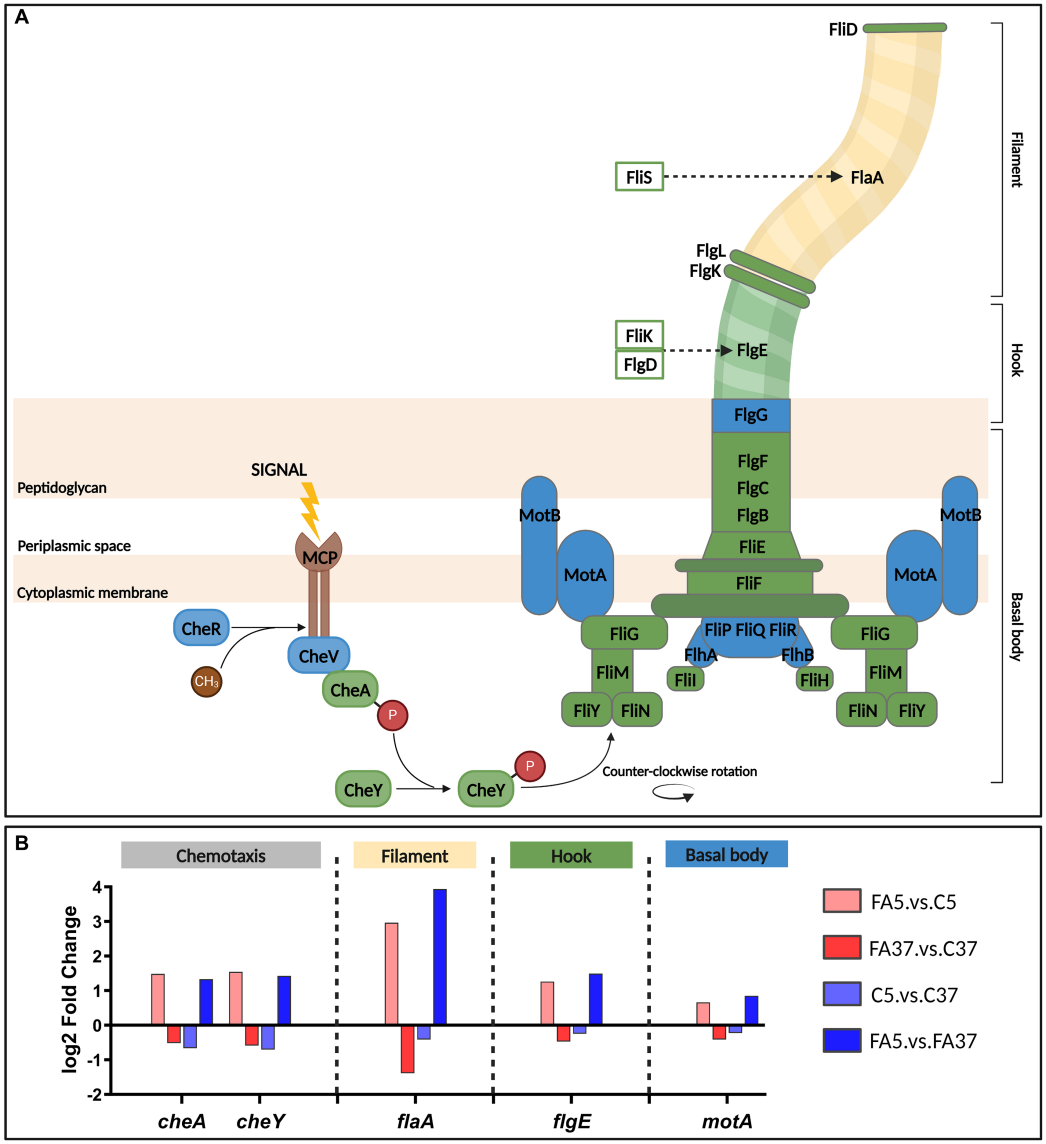
Chemotaxis and flagellar assembly in *L. monocytogenes* and the transcriptome of key genes at 5°C or 37°C with #C18:1 or without. **(A)** Chemotaxis signal transduction and organization of flagellar proteins [adapted from Liu and Ochman (2007), Porter et al. (2011), and Cheng et al. (2018)]. Proteins from operon 111, 112 and FlaA are in blue, green and yellow, respectively. **(B)** Differential expression (log2 fold change) of genes of interest. The figure was created with BioRender.com.

56.4% of DE5 genes are involved in cell motility, in particular in flagellar assembly. Genes involved in flagellum metabolism are regulated by the TCS CheAY and the response regulator DegU. They are distributed in two large operons (operon 112 in DE5 and operon 111 not classified) with the *flaA* gene encoding the flagellin protein in between (Figure 3A). The sensory *cheA* and its corresponding response regulatory *cheY* were down-regulated at low temperature (log2FC of −0.67 and −0.70, respectively), whereas they were up-regulated with #C18:1 at 5°C with a log2FC of 1.48 and 1.54, respectively (Figure 3B). Similarly, the genes of the operon 112 and *flaA* (Figure 3A, represented respectively in green and yellow), which are involved in the assembly of the filament, the hook, and the MS-ring of the basal body, were down-regulated at 5°C and up-regulated when #C18:1 was present (mainly *fliF*; *fliG*; *fliH*; *fliI*, and *flaA*). The genes of the operon 111 (Figure 3A, represented in blue) involved in the motor switch and the export apparatus were up-regulated only when temperature decreased in the presence of #C18:1 (FA5.vs.FA37).

46.7% of DE9 genes are involved in amino acid transport and metabolism. DE9 genes were up-regulated (log2FC >1) at 5°C, but the expression of these genes did not change significantly with #C18:1 at this temperature.

### 3.2 Deep assessment of the FASII pathway in the global transcriptome

**Figure 4:**
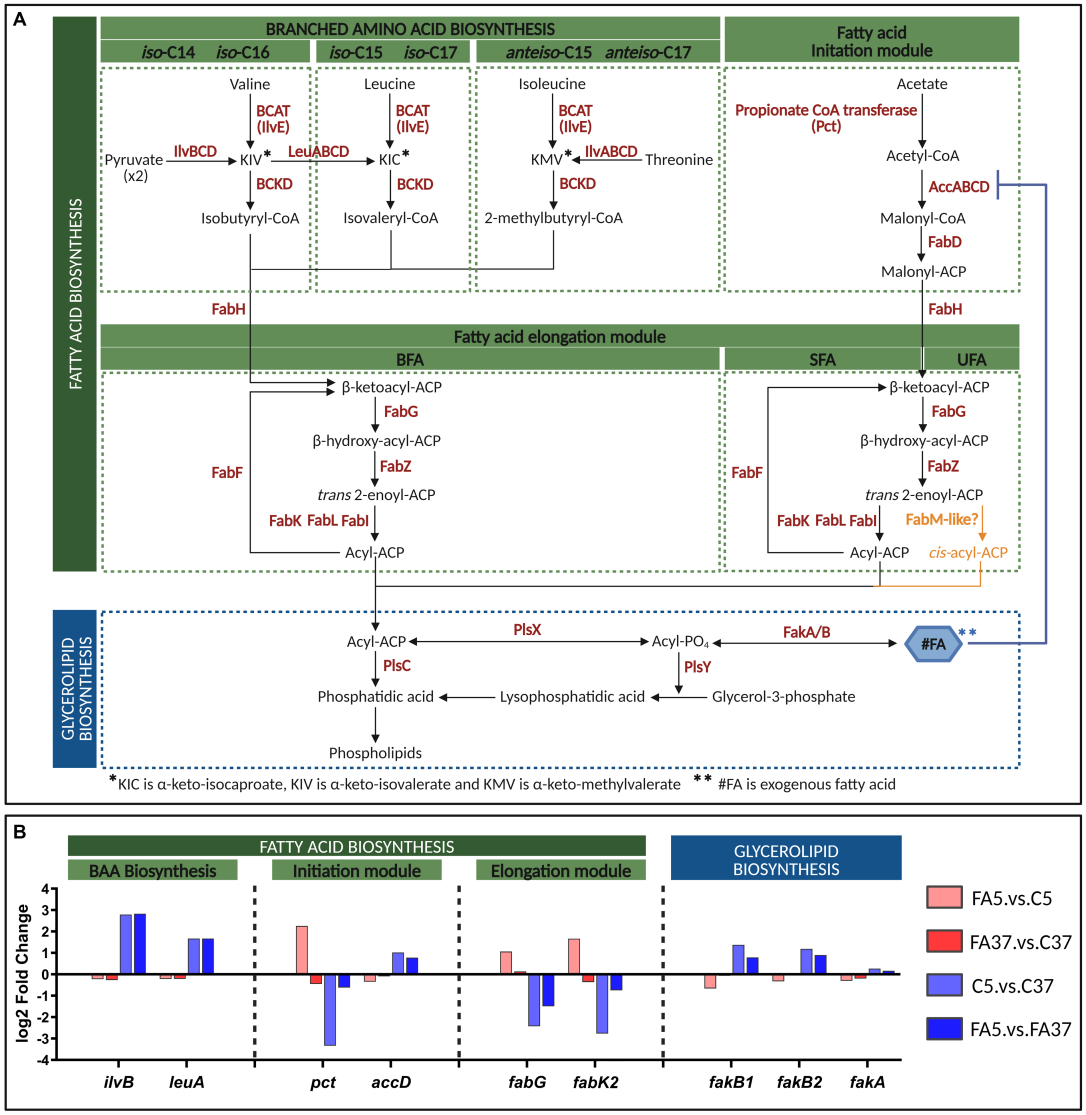
Fatty acid biosynthesis of *L. monocytogenes* and transcriptome of key genes at 5°C or 37°C with or without #C18:1. **(A)** Pathways of FASII and glycerolipid biosynthesis in *L. monocytogenes* [adapted from Parsons and Rock (2013) and Yao and Rock (2017)]. A hypothetical pathway for UFA synthesis is depicted in orange. **(B)** Differential expression (log2 fold change) of genes of interest. The figure was created with BioRender.com.

Here, we focused on the genes involved in the well-described lipid metabolism pathways leading to the synthesis of SFA and BFA (Figure 4A). Genes involved in BFA biosynthesis, forming operon 360 (*ilvABCDN* and *leuABCD*), were clustered in DE2. These genes were up-regulated at 5°C (C5.vs.C37) with a log2FC of 2.79 and 1.67 for *ilvB* and *leuA*, respectively (Figure 4B and Supplementary Table S2), but the presence of #C18:1 at the same temperature (FA5.vs.C5) did not induce significant differential expression (Figure 4B and Supplementary Table S2). No significant difference in gene expression was observed for *ilvE* and genes involved in the branched-chain α-ketoacid dehydrogenase complex formation, whatever the culture conditions (Supplementary Table S2). Among genes involved in the FASII initiation module, *pct* (*lmo2172*) was down-regulated at 5°C (log2FC = −3.34) but up-regulated with #C18:1 at the same temperature (log2FC = 2.26) (Figure 4B). In contrast, *accABCD* genes, involved in the formation of the ACC (acetyl-CoA carboxylase) complex, were up-regulated at low temperature (log2FC = 1.02 for *accD*), but not differentially expressed in the presence of #C18:1 at the same temperature (Figure 4B and Supplementary Table S2). Among genes involved in the elongation module of FA biosynthesis, two genes showed noticeable opposite differential expressions (Figure 4B). At low temperature, *fabK2* (*lmo2170*) and *fabG* (*lmo2175*) were down-regulated (log2FC = −2.77 and −2.43, respectively), while the addition of #C18:1 induced their up-regulation (log2FC = 1.66 and 1.06, respectively) (Figure 4B). Genes involved in #FA uptake, in particular the genes *fakB1* and *fakB2* encoding FA-binding proteins, were up-regulated at low temperature (log2FC = 1.38 and 1.19, respectively), while their differential expressions in the presence of #C18:1 at the same temperature were down-regulated (*fakB1*) or not significant (*fakB2*) (Figure 4B). No significant differences were observed for the gene *fakA* encoding the FA kinase, whatever the conditions (Figure 4B). Genes involved in glycerolipid biosynthesis, *plsX*, *plsY*, and *plsC*, showed no variation in their expression profile, whatever the conditions (Figure 4B).

### 3.3 Expression profile of five genes of interest by RT-qPCR

To extend the results of the global transcriptome analysis, five genes whose expression was impacted by oleic acid supplementation at low temperature (inverse differential expression between C5.vs.C37 and FA5.vs.C5) were selected for targeted expression analysis using RT-qPCR. These genes are involved in FA biosynthesis (*pct*, *fabG*, and *fabK2*) and chemotaxis and flagellar assembly (*cheY*, *flaA*). Their levels of relative expression were compared at low temperature in the presence of different #FA (#C14, #C18:1, or #C18:2) and at different time points in the growth phase (T1, T2, T3). First of all, the RT-qPCR results are consistent with the global transcriptome analysis (in presence of #C18:1 at T1).

**Figure 5:**
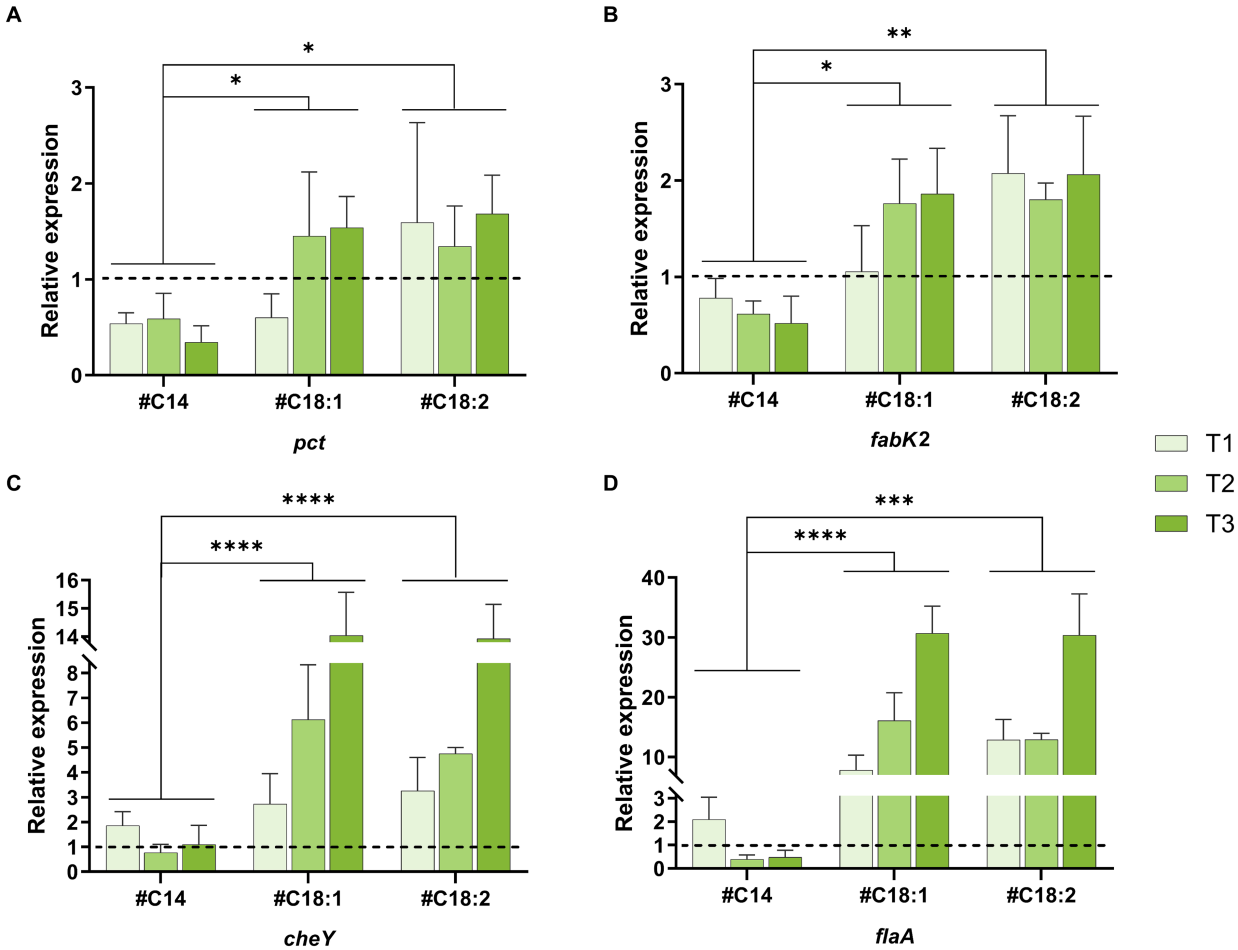
Quantitative RT-qPCR analysis of four genes of interest. Relative expression of *pct* **(A)**, *fabK2* **(B)**, *cheY* **(C)** and *flaA* **(D)** of *L. monocytogenes* at 5°C with #C14, #C18:1, #C18:2 at time points T1, T2, T3. The control is fixed to a relative expression level of 1 (dashed black line). Mean and standard deviation are represented (*n* = 3 to 6).

The relative expressions (2^−ΔΔCT^) of *pct* in comparison to the control was higher than 1 with #C18:1 and #C18:2 (except T1 in the presence of #C18:1) while it is lower than 1 with #C14 (Figure 5A). No significant difference was observed between cultures with the two #UFA. Furthermore, the difference in relative expression between cultures with #UFA and #C14 was significant (#C18:1: *p*-value = 0.0339 and #C18:2: *p*-value = 0.0234). The relative expressions (2^−ΔΔCT^) of *fabK2* in comparison to the control was higher than 1 with #C18:1 and #C18:2 while it is lower than 1 with #C14 (Figure 5B). No significant difference was observed between the two cultures with #UFA. Furthermore, the difference in relative expression between cultures with #UFA and cultures with #C14 was significant (#C18:1; *p*-value = 0.0339 and #C18:2; *p*-value = 0.0043). *fabG* was up-regulated with #C18:1 at T1 compared to the control (2^−ΔΔCT^ = 1.35), but not in all other conditions (data not shown).

The relative expressions (2^−ΔΔCT^) of *cheY* and *flaA* in comparison to the control were higher than 1 when the medium was supplemented with #C18:1 or #C18:2 (Figures 5C,D). For each #UFA, the relative expressions of *cheY* and *flaA* significantly increased over time (from T1 to T3) and reached more than 14 and 30, respectively. No significant difference was observed between cultures with the two #UFA. In the presence of #C14, a significant difference in the relative expression level of both genes was only observed with the control at T1. The difference in relative expression between cultures with #UFA and #C14 was overall highly significant (*p*-value <0.0001). Unlike *flaA* up-regulated at all time points, the relative expression level of *cheY* depended on the growth phase. Its relative expression at T3 with #C18:1 or #C18:2 was significantly different from the other growth phases (#C18:1: *p*-value <0.0001 and 0.004, respectively, and #C18:2: *p*-value <0.0001).

### 3.4 Membrane fatty acid profile

**Figure 6:**
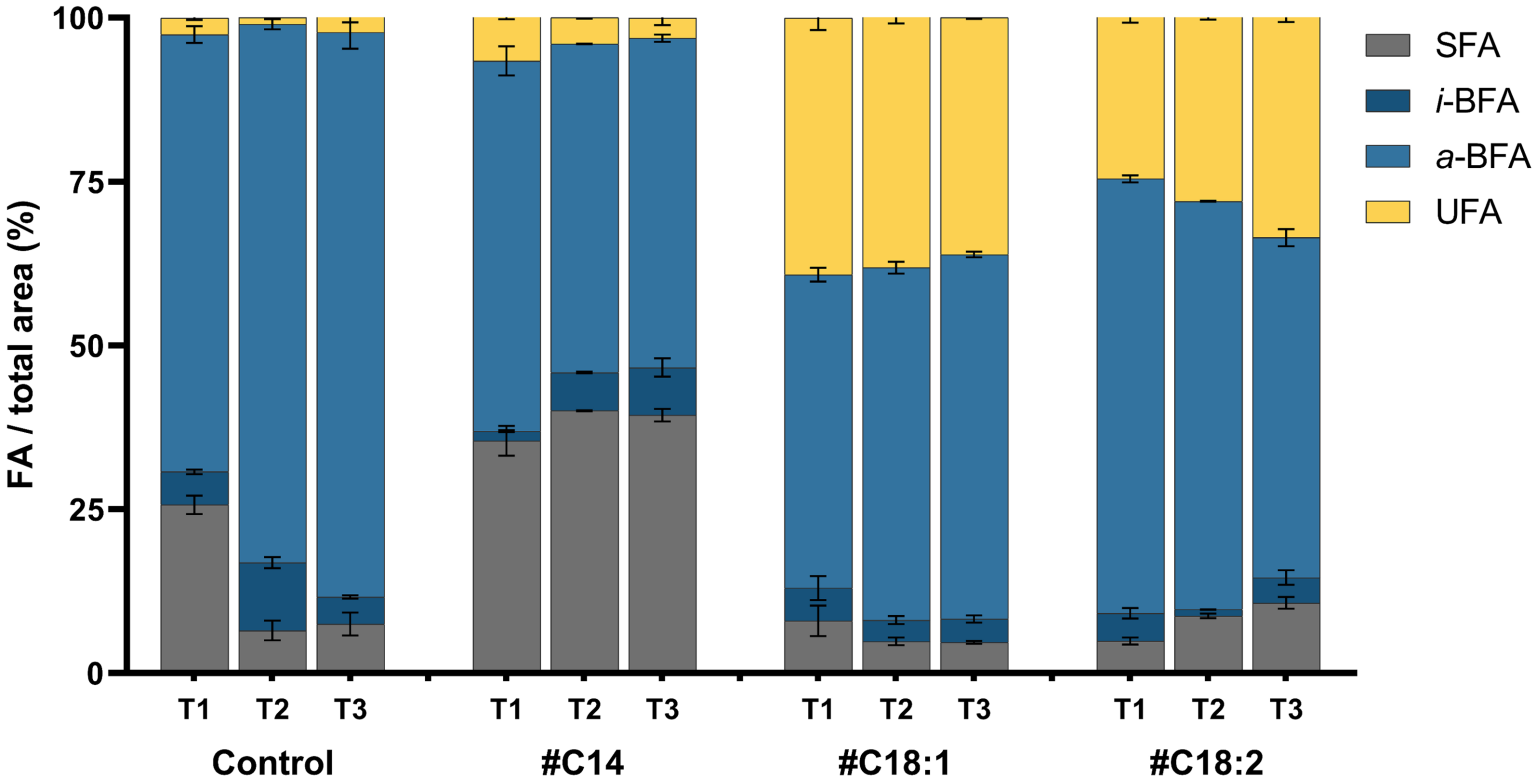
Fatty acid composition expressed in four FA categories (SFA, *i-*BFA, *a-*BFA and UFA) of *L. monocytogenes* at 5°C with #C14, #C18:1, #C18:2 or without (Control) at time points T1, T2, T3. Mean and standard deviation are represented (*n* = 3).

The FA profiles of Lm208 were compared at low temperature in the presence of different #FA (#C14, #C18:1, or #C18:2) and at different time points in the growth phase (T1, T2, T3). The FA composition of Lm208 without #FA was predominantly composed of BFA with 71.7, 92.5, and 90.3% of the total FA at T1, T2, and T3, respectively. The most abundant *a*-BFA were *a*-C15:0 (61.5% average over all the growth phases) and *a*-C13:0 (16.2% average over all the growth phases). The proportion of total SFA was significantly lower at T2 (6.5%) and T3 (7.5%) in comparison to T1 (25.7%). The high proportion at T1 was due to a high proportion of C16:0 (11.6% at T1 versus 3.7 and 4.5% at T2 and T3, respectively). Total UFA represented less than 3% of membrane composition whatever the growth phase (Figure 6). In the presence of #C14, the level of SFA in the membrane increased significantly with a level of incorporation of 9.7, 33.5, and 31.9% at T1, T2, and T3, respectively, compared to the control. The membrane of #C14 cultures contained 31.9% of C14 versus 4.2% in the control at T1. No significant differences were observed between the different time points of the growth phase (Figure 6). In the presence of #C18:1, the proportion of UFA was significantly higher compared to the control (39.2, 36.8, and 36.1% at T1, T2, and T3, respectively). The increased proportion was almost exclusively due to the presence of #C18:1 in the membrane. The incorporation of #C18:1 did not significantly change over time. With #C18:2, the results had the same trend as those obtained with #C18:1, but the incorporation of #C18:2 significantly increased with time (21.6%, 24.4, and 30.1% at T1, T2, and T3, respectively) (*p*-value <0.0001 between T1 and T3).

### 3.5 Cell and flagella morphologies by TEM

**Figure 7:**
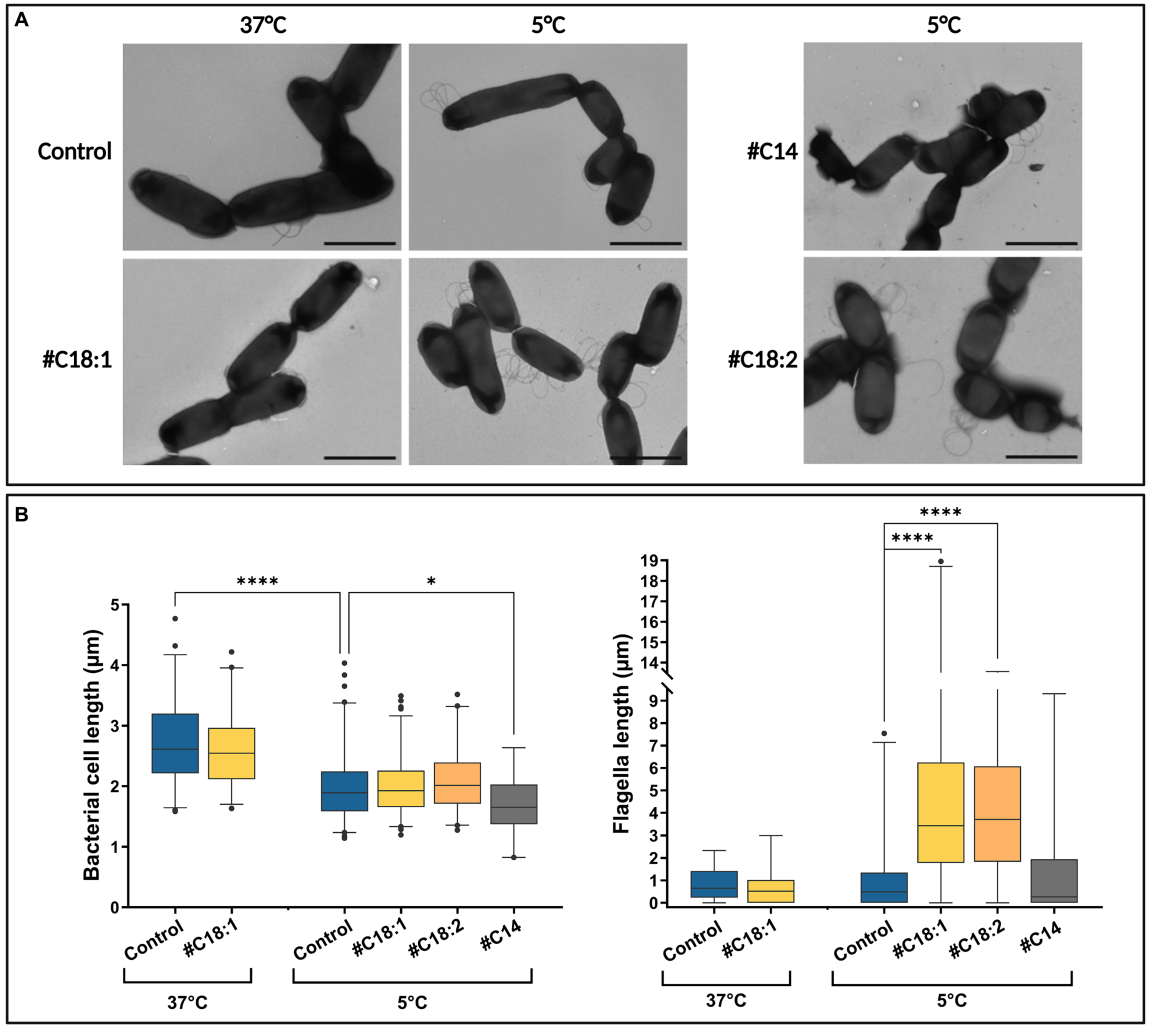
**(A)** TEM images and **(B)** bacterial cell and flagellar lengths of *L. monocytogenes* grown at 5°C or 37°C with #C18:1, #C18:2, #C14 or without (scale bar = 2 μm).

Cell and flagella morphologies were compared at 5°C with #C14, #C18:1, or #C18:2 or without #FA at the three time points, and at 37°C with #C18:1 or without #FA at T1. Bacterial cell and flagellar lengths per bacterium were measured in each condition from an overall number of more than 190 images. One representative TEM image of each condition is represented in Figure 7A. For all culture conditions, the time point in the growth phase did not indicate quantitative differences in bacterial and flagellar lengths per bacterium (data not shown). Without #FA, bacterial cell length significantly decreased at 5°C when compared to 37°C, while flagellar length per bacterium remained similar. With #C18:1, bacterial length was similar to the control at each temperature (Figure 7B). Conversely, flagella per cell were significantly longer with #C18:1 at 5°C but not at 37°C. With #C18:2, the results were similar to those with #C18:1 at 5°C. Moreover, many flagella form multiple loops in the images of the cultures grown with each #UFA at low temperature. There was also high variability in terms of flagella length when #UFA were present. With #C14 at 5°C, bacterial length was significantly shorter than the control, but the flagellar length was similar (Figure 7B).

## 4 Discussion

In our previous paper (Touche et al., 2023), we demonstrated that exogenous UFA (#UFA), but not exogenous SFA (#SFA), are growth promoters of *Listeria monocytogenes* at low temperature. We also showed that # UFA and #SFA are both over-incorporated at 5°C in comparison with 37°C. In the present study, we aimed to understand the impact of #FA on the behavior of *L. monocytogenes* at low temperature using untargeted and targeted transcriptomics together with the characterization of membrane FA profiles, and cell and flagellar morphologies.

Our results confirm that *L. monocytogenes*, like numerous other bacteria, can incorporate #FA into their membrane. Moreover, we showed here that, when *L. monocytogenes* was grown in the presence of #C14, #C18:1, or #C18:2, the three FA were highly incorporated since the first time point (mid-exponential phase). Bacterial FA profiles did not differ significantly at the subsequent time points of the growth phase (T2 and T3) in the presence of #C14 or #C18:1. The genes encoding FakB1 and FakB2, the FA-binding proteins for SFA and mono-UFA, are up-regulated at low temperature, but the presence of #C18:1 did not induce the increase of expression we could expect according to the corresponding incorporation. These results suggest that the up-regulation of *fakB1* and *fakB2* probably occurs at the early stage of the exponential growth phase, which is consistent with the early FA incorporation and with the changes in growth rate starting from the very beginning of growth (Touche et al., 2023). Furthermore, we can notice that the incorporation of #C18:2 at 5°C significantly increases with time as shown in the bacterial FA profiles. A *fakB3-like* gene encoding for a poly-UFA binding protein probably exists due to the ability of the strain to incorporate #C18:2. Nevertheless, the dynamics of its expression are still unknown because we did not identify it in the genome of Lm208 by BLAST and sequence comparison.

We also analyzed the impact of #FA on the regulation of the FA metabolism. The untargeted transcriptomic analysis showed that genes involved in FASII are greatly affected by both temperature and the presence of #C18:1. Genes involved in BFA synthesis (*ilvABCDN*, *leuABCD*) are up-regulated at low temperature (Figure 4 and Supplementary Table S2). The corresponding over-synthesis of BFA at the expense of SFA when temperature decreases is very well described in the literature (Chihib et al., 2003; Touche et al., 2023). Moreover, some genes involved in FA metabolism are down-regulated at low temperature but up-regulated with #C18:1, especially *pct* (*lmo2172*), encoding propionate-CoA-transferase, which is responsible for the first step of the initiation module of straight chain FA, and *fabK2* involved in FA *de novo* synthesis. The targeted transcriptomic of *pct* and *fabK2* conducted at low temperature with another #UFA (#C18:2) and one SFA (#C14) showed that both #UFA induced their up-regulation but not #SFA. This highlights the specificity of the regulation of these genes according to the nature of exogenous FA. *pct* is involved at the very beginning of the initiation module of FASII, but it is also involved in other pathways of bacterial synthesis, such as pyruvate metabolism. It is thus quite difficult to draw more hypotheses about its involvement in the uptake of #UFA. *fabK* regulates *de novo* synthesis of SFA and BFA in the membrane. Two *fabK* were identified in Lm208. Only *fabK2 (lmo2170)* expression was modulated according to the type of #FA: the presence of #SFA and #UFA in the medium induces the decrease and increase of *fabK2* expression, respectively. *fabK1 (lmo0814)* expression is only modulated by the temperature. In *Streptococcus pneumoniae*, it was shown that FabK competes with FabM for the same substrate (*trans*-2-decenoyl-ACP) to modulate the ratio of UFA/SFA in the membrane (Parsons and Rock, 2013). *fabK* expression has to be weakened to allow UFA synthesis to proceed (Dong and Cronan, 2022). Here, even if no *fabM* and *fabN* coding sequences or corresponding homologous protein sequences were not found in the *L. monocytogenes* genome (see Supplementary Table S2), we hypothesize that Lm208 possesses a *fabM/N-like* gene for UFA *de novo* synthesis because its FA membrane profile contains UFA when grown without #UFA (Touche et al., 2023; this study). We also hypothesize that *fabM/N* could compete with *fabK2* for modulating the ratio SFA/UFA in the membrane, according to the type of #FA available in its environment. Similarly, Zheng et al. (2005) showed that some #FA, but not all, inhibited FabI in *S. aureus* at 37°C. FabI, similarly to FabK, is an enoyl-ACP reductase that catalyzes the final step of the chain elongation process of the FASII. The modulation between the two pathways for SFA and UFA syntheses does not exist in *S. aureus* which cannot synthesize UFA. #UFA, but not #SFA, inhibited FabI, increased the fluidity of the *S. aureus* membrane, and showed antibacterial activity at 37°C (Zheng et al., 2005). The temperature probably plays here a very important role in the regulation of FASII pathways in the presence of #FA because of its direct impact on membrane fluidity.

In addition, in several bacterial strains, UFA are produced by a post-synthesis mechanism using a desaturase system. In *B. cereus*, two desaturases have been identified, DesA and DesB, which catalyze the formation of double bonds in the acyl chain of FA (Diomandé et al., 2015). To achieve double bond formation, desaturases recruit and activate molecular oxygen with the use of an active-site di-iron cluster (Diaz et al., 2002). No desaturase was found in the genome of Lm208 (Supplementary Table S2), but our results show that genes involved in iron uptake, such as *fhuCBG* and *tatAC*, are up-regulated when temperature decreases and down-regulated with #C18:1 at low temperature. This is compatible with the hypothesis, already suggested by Hingston et al. (2017) and Touche et al. (2023), that *L. monocytogenes* could possess an iron-dependent desaturase-like system which is activated by low temperature, but not when UFA are incorporated in the membrane.

Interestingly, we shed light on an unexpected connection between the presence of #UFA at low temperature and the expression of chemotaxis and flagellar assembly genes. At low temperature without #FA, most chemotaxis and flagellar assembly genes are not differentially expressed (*flaA*, operon 111 and 114) or down-regulated (operon 112), as already observed by Hingston et al. (2017). The impact of the temperature on *L. monocytogenes* is well known, this bacterium being motile below 25°C but not at 37°C (Liu et al., 2002; ANSES, 2020). The presence of #C18:1 at low temperature leads to the up-regulation of all genes in operon 112, *motA*, *gamR*, *cheV* in operon 111, and *flaA*, in association with the appearance of a remarkable bacterial phenotype trait, being numerous and long-looped flagella. More specifically, the targeted transcriptomic analysis revealed that *cheY* and *flaA* were also up-regulated in the presence of another #UFA (#C18:2), but not in the presence of #SFA (#C14). TEM observations confirm the increase in flagellar length per bacterium in the presence of #UFA, but not with #SFA. In addition, this phenotype appeared with #C18:1 only at 5°C but not at 37°C. We thus hypothesize a role of #UFA in the regulation of genes involved in motility and flagella biosynthesis at low temperature. In *L. monocytogenes*, ∆*cheAY* and ∆*degU* mutants are respectively non-motile and non-flagellated (Dons et al., 2004; Williams et al., 2005). As in most firmicutes, DegU is the temperature-response regulator of motility and chemotaxis in *L. monocytogenes*. When active, at lower temperature than 25°C, DegU directly represses its own expression by binding its promoter and activates chemotaxis and flagellar motility gene expression by binding the upstream region of operon 111 and the *flaA* gene (Knudsen et al., 2004; Williams et al., 2005; Gueriri et al., 2008). Gueriri et al. (2008) have shown that *degU* also controls the expression of two uncharacterized operons (operons 114 and 295) each of them encoding one methyl-accepting chemotaxis protein (MCP). In our results, these two operons, like operon 112 and *flaA*, are up-regulated in the presence of #C18:1 at low temperature. MCP are transmembrane proteins able to sense chemical and physical signals and are the starting point of the phosphorylation cascade activating the response regulator of a two-component system (TCS). The MCP of operon 114 (*lmo0723*) has been reported as part of CheAY TCS in which it transduces the external signal from extracellular to cytoplasmic histidine kinase (Lacal et al., 2010; Casey et al., 2014). Nevertheless, the cognate DegS kinase of DegU is noticeably absent in the genome of *L. monocytogenes* (Gueriri et al., 2008). The signal sensing system and the nature of the signal remain unknown. We hypothesize here that MCP in operon 295 (*lmo1699*) could be involved in DegU phosphorylation and that the presence of #UFA could be a chemical signal that activates the system. The chemotaxis signal detected by MCP could be due either to the physical state of the membrane or the specific chemical signal of #UFA.

## 5 Conclusions and perspectives

In the present study, we first demonstrated that oleic acid supplementation at low temperature significantly impacts the gene expression of *L. monocytogenes*. Analysis of the expression of genes involved in FA biosynthesis suggests the presence of two potential UFA biosynthesis pathways in *L. monocytogenes*. To identify the genes that could support our hypotheses, such as a *fabM-*like, *des-*like or *fakB3-*like, a bank of deletion mutants will be constructed and submitted to different FA types at low temperature. In addition, we demonstrate that *L. monocytogenes* grown in the presence of UFA but not SFA at low temperature overproduces flagella. These results raise new questions to be addressed, such as the ability of these bacteria to adhere to inert surfaces and produce biofilms in food processing environments. Moreover, as we noted heterogeneity in terms of flagellar length when #UFA are present in the environment, it could be interesting to monitor the expression level of genes of interest at the single-cell level with fluorescent reporters to evaluate the heterogeneity of gene expression in the whole population. Addressing these new questions will help improve understanding of the behavior of *L. monocytogenes* at low temperature and thus help find ways to control this pathogen in foods and food environments.

## Data Availability

The datasets presented in this study can be found in online repositories. The names of the repository/repositories and accession number(s) can be found in the article/Supplementary material.

## Footnotes

^1^https://biocyc.org/

^2^https://www.ncbi.nlm.nih.gov/tools/primer-blast/
