# Supplementary figures and images for: Deciphering the impact of exogenous fatty acids on Listeria monocytogenes at low temperature by transcriptome analysis
Source: Front Microbiol. 2024 Sep 4;15:1441784. doi: 10.3389/fmicb.2024.1441784 (PMC11426360; doi:10.3389/fmicb.2024.1441784)

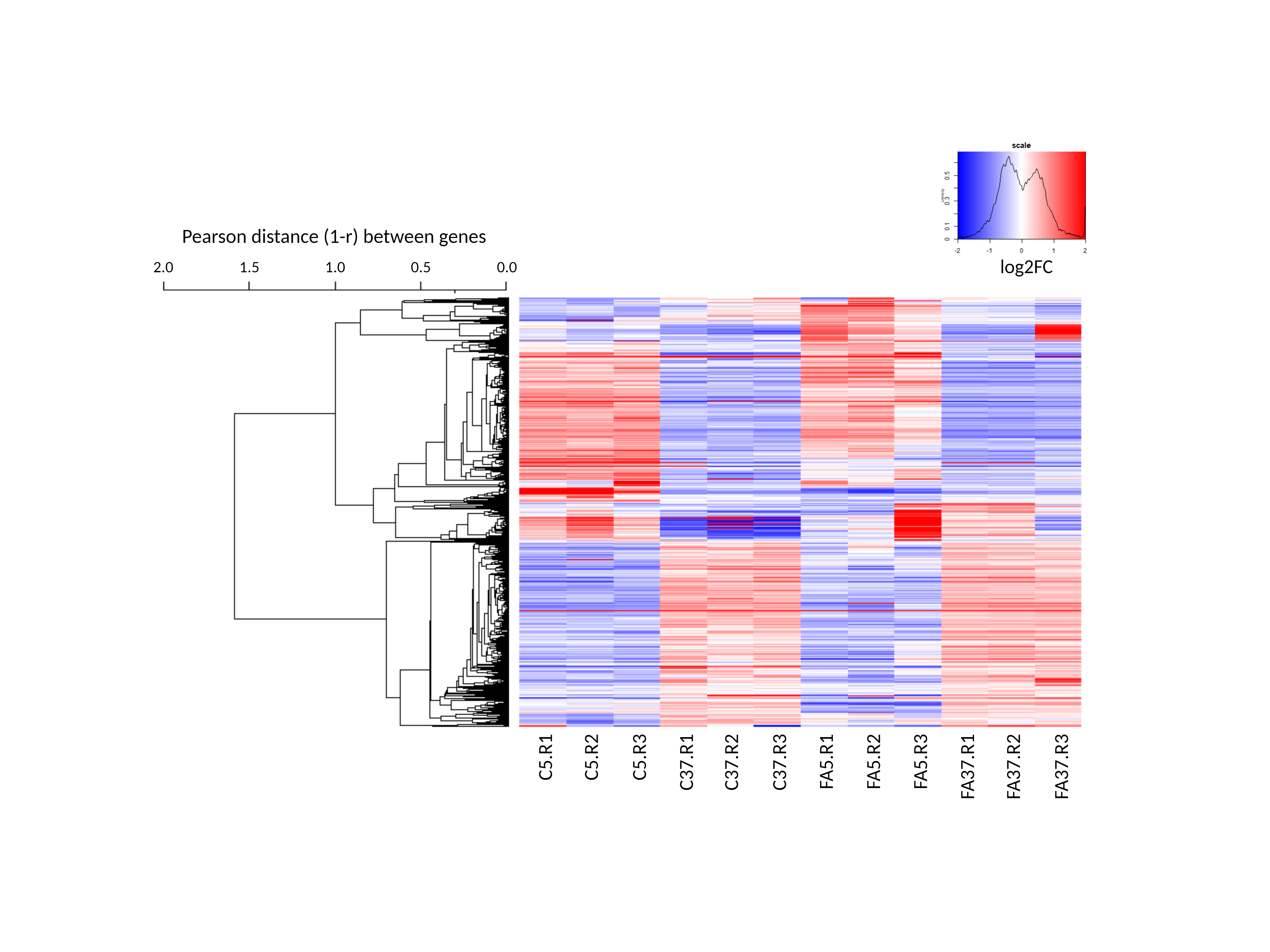

Supplement: Supplementary file 1 [file Image_1.JPEG]
