# Supplementary material for: Deciphering the impact of exogenous fatty acids on Listeria monocytogenes at low temperature by transcriptome analysis
Source: Front Microbiol. 2024 Sep 4;15:1441784. doi: 10.3389/fmicb.2024.1441784 (PMC11426360; doi:10.3389/fmicb.2024.1441784)
Supplement: Supplementary file 2 [file Table_1.DOCX]

**Table S1:** Functional category distribution for each gene expression cluster. Gene expression clusters are named from DE1 to DE12 by decreasing size and contain more than 10 genes. Functional categories are derived from the Listeriomics database. Some genes are involved in several functional categories, which is why the sum of percentages may exceed 100%.

| Functional categories | **DE1** | **DE2** | **DE3** | **DE4** | **DE5** | **DE6** | **DE7** | **DE8** | **DE9** | **DE10** | **DE11** | **DE12** |
| --- | --- | --- | --- | --- | --- | --- | --- | --- | --- | --- | --- | --- |
| Amino acid transport and metabolism | 6.63 | 16.67 | 5.19 | 0.00 | 3.13 | 0.00 | 6.90 | 13.33 | 46.67 | 0.00 | 7.69 | 18.18 |
| Carbohydrate transport and metabolism | 13.79 | 10.26 | 14.29 | 2.90 | 6.25 | 0.00 | 6.90 | 26.67 | 0.00 | 0.00 | 46.15 | 18.18 |
| Cell cycle control, mitosis and meiosis | 1.33 | 0.32 | 0.00 | 2.90 | 0.00 | 0.00 | 0.00 | 0.00 | 0.00 | 0.00 | 0.00 | 0.00 |
| Cell motility | 1.06 | 0.32 | 0.00 | 1.45 | 56.25 | 3.23 | 0.00 | 0.00 | 0.00 | 0.00 | 0.00 | 0.00 |
| Cell wall/membrane biogenesis | 4.77 | 1.28 | 6.49 | 14.49 | 0.00 | 3.23 | 10.34 | 0.00 | 0.00 | 0.00 | 7.69 | 0.00 |
| Coenzyme transport and metabolism | 1.33 | 2.88 | 2.60 | 5.80 | 0.00 | 0.00 | 10.34 | 0.00 | 0.00 | 0.00 | 0.00 | 0.00 |
| Defense/virulence mechanisms | 2.65 | 2.56 | 1.30 | 7.25 | 0.00 | 0.00 | 6.90 | 0.00 | 0.00 | 0.00 | 0.00 | 0.00 |
| Energy production and conversion | 4.51 | 1.60 | 7.79 | 2.90 | 0.00 | 0.00 | 3.45 | 0.00 | 33.33 | 0.00 | 7.69 | 18.18 |
| General function prediction only | 12.20 | 16.03 | 15.58 | 8.70 | 9.38 | 3.23 | 0.00 | 20.00 | 0.00 | 0.00 | 15.38 | 18.18 |
| Inorganic ion transport and metabolism | 5.31 | 4.49 | 5.19 | 30.43 | 3.13 | 0.00 | 3.45 | 13.33 | 0.00 | 0.00 | 0.00 | 36.36 |
| Intracellular trafficking and secretion | 1.06 | 0.32 | 1.30 | 4.35 | 15.63 | 0.00 | 3.45 | 0.00 | 0.00 | 0.00 | 0.00 | 0.00 |
| Lipid transport and metabolism | 0.53 | 0.32 | 1.30 | 2.90 | 0.00 | 0.00 | 0.00 | 13.33 | 0.00 | 0.00 | 0.00 | 0.00 |
| Nucleotide transport and metabolism | 5.04 | 0.96 | 0.00 | 0.00 | 0.00 | 0.00 | 0.00 | 6.67 | 0.00 | 0.00 | 0.00 | 0.00 |
| Posttranslational modification, protein turnover, chaperones | 4.24 | 1.60 | 1.30 | 4.35 | 3.13 | 0.00 | 3.45 | 6.67 | 0.00 | 0.00 | 0.00 | 0.00 |
| Replication, recombination and repair | 3.45 | 2.24 | 0.00 | 0.00 | 0.00 | 0.00 | 3.45 | 0.00 | 0.00 | 0.00 | 0.00 | 0.00 |
| Secondary metabolites biosynthesis, transport and catabolism | 1.59 | 1.60 | 3.90 | 0.00 | 0.00 | 0.00 | 0.00 | 0.00 | 33.33 | 0.00 | 7.69 | 0.00 |
| Signal transduction mechanisms | 3.98 | 4.81 | 9.09 | 1.45 | 18.75 | 3.23 | 0.00 | 6.67 | 0.00 | 0.00 | 0.00 | 0.00 |
| Transcription | 8.75 | 13.46 | 5.19 | 2.90 | 3.13 | 3.23 | 3.45 | 0.00 | 0.00 | 0.00 | 0.00 | 0.00 |
| Translation | 4.24 | 2.24 | 1.30 | 0.00 | 0.00 | 3.23 | 0.00 | 0.00 | 0.00 | 0.00 | 0.00 | 0.00 |
| Function unknown / Not in COGs / NA | 26.79 | 31.41 | 33.77 | 20.29 | 25.00 | 80.65 | 44.83 | 20.00 | 13.33 | 100.00 | 23.08 | 27.27 |

Table S2: Differential expression (log2 fold change) and associated gene expression clusters (DE) of *Listeria monocytogenes* Lm208 with #C18:1 or without at 5°C and 37°C. The four pairwise comparisons were analyzed: “FA5.vs.C5”; “FA37.vs.C37”; “C5.vs.C37”; “FA5.vs.FA37” with “FA” and “C” corresponding to #C18:1 and to control conditions without #FA, respectively, and  “5” and “37” refer to the culture temperature in °C. The code summarizing up- and down-regulated gene when q-value < 0.05: “U+” if log2FC > 1, “u” if 0.5< log2FC < 1, “D-” if log2FC < -1, “d” if -1 < log2FC < -0.5, “~” in all other cases.

Figure S1: Global heatmap representation of the relative variations of expression level between each biological replicate in each culture condition for differentially expressed genes. C and FA refer to control cultures and cultures grown with #C18:1, 5 and 37 refer to the culture temperature, and R1, R2, R3 refer to biological replicates. Differentially expressed genes (1068 genes) were clustered by their expression profile across all samples (dendrogram on the left side). Up‑regulated genes are reported in red (q ≤ 0.05 and log2FC ≥ 1) and down‑regulated genes in blue (q ≤ 0.05 and log2FC ≤ ‑1).
